# Dioscin Protects against Cisplatin-Induced Acute Kidney Injury by Reducing Ferroptosis and Apoptosis through Activating Nrf2/HO-1 Signaling

**DOI:** 10.3390/antiox11122443

**Published:** 2022-12-11

**Authors:** Shuang Wang, Yingce Zheng, Shengzi Jin, Yunwei Fu, Yun Liu

**Affiliations:** 1 College of Veterinary Medicine, Northeast Agricultural University, Harbin 150030, China; 2 College of Life Science, Northeast Agricultural University, Harbin 150030, China; 3 Northeast Agricultural University Animal Hospital, Harbin 150030, China; 4 Heilongjiang Province Key Laboratory of Pathogenic Mechanism for Animal Disease and Comparative Medicine, College of Veterinary Medicine, Northeast Agricultural University, Harbin 150030, China

**Keywords:** acute kidney injury, dioscin, oxidative stress, apoptosis, ferroptosis, Nrf2/HO-1 signaling

## Abstract

Acute kidney injury (AKI) is a clinical syndrome with high morbidity and mortality worldwide, and there is currently no effective means to prevent it. Dioscin is naturally present in the dioscoreaceae plants and has antioxidant and anti-inflammatory effects. Here, we found that dioscin is protective against cisplatin-induced AKI. Pathological and ultrastructural observations revealed that dioscin reduced renal tissue lesions and mitochondrial damage. Furthermore, dioscin markedly suppressed reactive oxygen species and malondialdehyde levels in the kidneys of AKI rats and increased the contents of glutathione and catalase. In addition, dioscin dramatically reduced the number of apoptotic cells and the expression of pro-apoptotic proteins in rat kidneys and human renal tubular epithelial cells (HK2). Conversely, the protein levels of anti-ferroptosis including GPX4 and FSP1 in vivo and in vitro were significantly enhanced after dioscin treatment. Mechanistically, dioscin promotes the entry of Nrf2 into the nucleus and regulates the expression of downstream HO-1 to exert renal protection. However, the nephroprotective effect of dioscin was weakened after inhibiting Nrf2 in vitro and in vivo. In conclusion, dioscin exerts a reno-protective effect by decreasing renal oxidative injury, apoptosis and ferroptosis through the Nrf2/HO-1 signaling pathway, providing a new insight into AKI prevention.

## 1. Introduction

Acute kidney injury (AKI) is a clinically common syndrome associated with a dramatic decline in renal function, which leads to high morbidity and mortality. AKI can also develop into chronic kidney disease, thereby affecting patients’ quality of life [1]. The predisposing factors for AKI are diverse and include chemical drugs, ischemia, and toxins. Moreover, AKI surgical procedures are complicated, which increase the risk of death among patients [2]. The current treatment of AKI is mainly based on renal replacement therapy, such as intermittent hemodialysis, which is relatively expensive. Therefore, developing economic, safe, and effective methods to prevent and treat AKI is essential.

Cisplatin (CDDP) is a well-known anti-tumor chemical drug, and due to its severe nephrotoxicity, it is widely used in the laboratory for establishing AKI animal models [3]. Various unfavorable factors, such as chemical drugs, may lead to aberrant apoptosis by inducing the release of lethal factors, such as cytochrome C (CytC) and cysteinyl aspartate specific proteinase 3 (Caspase3) in renal tubular epithelial cells, eventually damaging the renal tissues [4]. Unlike apoptosis, ferroptosis is an iron-dependent form of regulated cell death, and it is recognized that peroxidative damages of polyunsaturated-fatty-acid-containing phospholipids is the cause of ferroptosis [5]. Glutathione peroxidase 4 (GPX4) is an inhibitory protein of lipid peroxidation which can degrade lipid peroxides to improve ferroptosis. In addition, as a co-factor for GPX4 to catalyze the conversion of peroxides to water or the corresponding alcohols, glutathione (GSH) deficiency would directly lead to inactivation of GPX4, ultimately resulting in ferroptosis [6]. Historically, ferroptosis suppressor protein 1 (Fsp1) has been recognized as a mitochondrial apoptosis-inducing factor until Bersuker et al. discovered that it acts as a lipophilic free radical trapping antioxidant to reduce lipid peroxidation and thereby prevent ferroptosis [7]. Hu et al. found that after the intraperitoneal injection of the ferroptosis inhibitor ferrostatin-1 (Fer-1), cell death in the kidney tissue of AKI mice was reduced, suggesting that ferroptosis is involved in AKI [8]. Furthermore, many studies have confirmed that the oxidation and anti-oxidation balance in the renal tissue is disrupted during AKI. A decrease in the level of antioxidant substances, such as GSH, leads to the accumulation of oxidation products, such as reactive oxygen species (ROS), and malondialdehyde (MDA), that cannot be cleared in time, thereby causing oxidative stress in the renal tissues [9]. Therefore, apoptosis, ferroptosis, and oxidative stress are crucial in alleviating AKI.

Heme oxygenase 1 (HO-1) plays a protective role when the body is exposed to ex-ternal stimuli, such as ROS, inflammation, and hypoxia [10,11]. Studies have illustrated that activation of the nuclear factor erythroid 2-related factor 2 (Nrf2)/HO-1 pathway may alleviate oxidative stress and confer protection to the heart, liver, and other tissues [12,13]. Researchers have also discovered that the Nrf2/HO-1 signaling pathway has anti-apoptosis and antioxidant effects on different tissues and cells. For instance, Dang et al. reported that alantolactone prevented cigarette smoke extract-induced human bronchial epithelial cell injury by activating the Nrf2/HO-1 pathway to suppress apoptosis related-genes (including caspase 3) and oxidative stress [14]. Interestingly, Kwon et al. reported that HO-1 accelerates erastin-induced ferroptotic cell death [15] because HO-1 can oxidize cellular heme to carbon monoxide (CO) and free ferrous iron (Fe^2+^) [16]. Therefore, it is necessary to further elucidate the exact role of the Nrf2/HO-1pathway in the cisplatin-induced AKI model, which may provide a potential therapeutic strategy to control AKI.

Dioscin is a natural steroidal saponin existing in Dioscoreae plants, and its antioxidant and anti-inflammatory and anticancer effects have been found [17,18]. Previous studies have demonstrated that dioscin improves lipopolysaccharide-induced mammary gland inflammation by reducing inflammasome activation and inhibiting inflammatory factor expression [19]. In a model of doxorubicin-induced cardiotoxicity, the administration of dioscin effectively inhibited the accumulation of ROS and MDA in cells and increased the levels of superoxide dismutase (SOD) and GSH, thereby protecting the myocardium from oxidative stress [20]. In addition, Li et al. found that dioscin ameliorated methotrexate-induced liver and kidney injury by decreasing oxidative stress, and in this process, dioscin enhanced the levels of GSH, Nrf2 and HO-1 in liver and kidney as well as repressed ROS and MDA [21]. Furthermore, a recent study showed that the oral administration of dioscin to diabetic rats significantly suppressed oxidative stress and inflammation in the kidneys of rats, and improved mitophagy and abnormal mitochondrial fission and fusion to arrest apoptosis, thereby alleviating kidney damage [22]. However, the effect and molecular mechanism of dioscin on AKI, especially from the perspective of ferroptosis, remains unclear. In this current study, we established an AKI model in vitro and in vivo by treating rats and human renal tubular epithelial cells (HK2) with cisplatin, and explored the protective effect of dioscin on kidneys from three aspects: oxidative stress, ferroptosis and apoptosis. Furthermore, we inhibited the expression of the key gene Nrf2 in vitro and in vivo to determine whether dioscin exerts a nephroprotective effect through the Nrf2/HO-1 signaling pathway. These findings provide new ideas for mitigating ferroptosis and a potential approach for the treatment of AKI.

## 2. Materials and Methods

### 2.1. Preparation of Animals and Samples Collection

**Figure 1:**
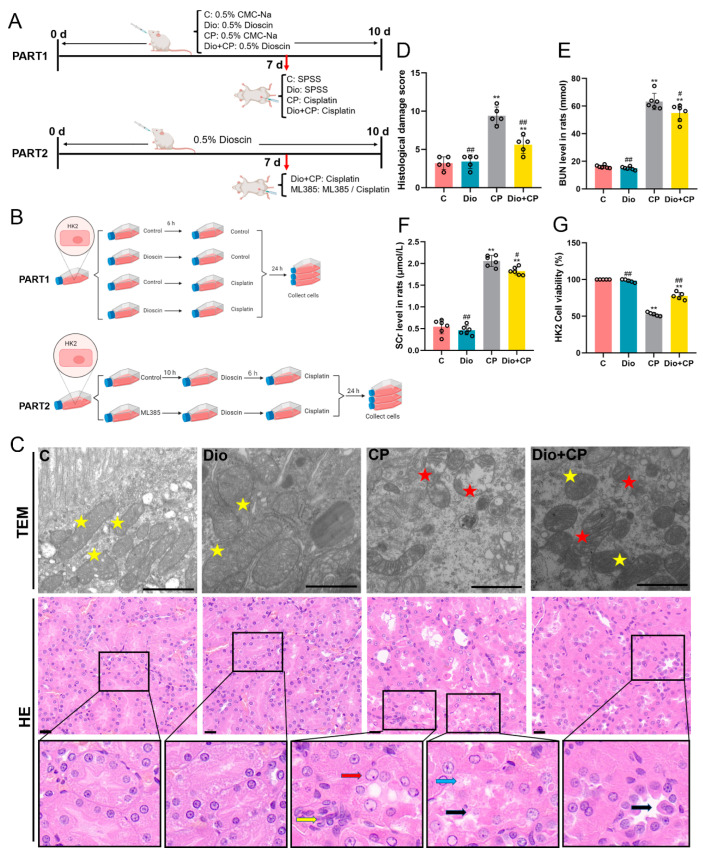
Dioscin relieves cisplatin-induced AKI. (**A**) Schematic representation of animal study design in the present study. SPSS, stroke-physiological saline solution. (**B**) Schematic diagram of cell experiment design. (**C**) Transmission electron microscope (scale bar = 1 μm) and H&E staining (scale bar = 20 μm) of rat kidney tissue (*n* = 5). Yellow pentagram, normal mitochondria; red pentagram, damaged mitochondria; red arrow, abnormal nucleus; yellow arrow, inflammatory cell infiltrated; blue arrow, renal tubular epithelial cell degeneration; black arrow, renal tubular epithelial cell desquamation. (**D**) Histological damage score. (**E**,**F**) Bun and SCr levels in rat serum (*n* = 6). (**G**) HK2 cell activity. C, control group; Dio, dioscin group; CP, cisplatin group; Dio + CP, dioscin + cisplatin group. Results are presented as Mean ± SD. Statistical significance was obtained by one-way ANOVA. ** *p* < 0.01 compared with C group. ^#^ *p* < 0.05, ^##^ *p* < 0.01 compared with CP group.

Six-week-old male Wistar rats (170–200 g) were obtained from Changsheng Biotechnology Co., Ltd. (Changchun, China), and all of them were fed under SPF-conditions. The rats were acclimatized to natural light/dark cycles at a controlled temperature of 22 + 2 °C with free access to food and water. The experiment was comprised of four groups: the C group (0.5% carboxymethyl cellulose sodium [CMC-Na], *n* = 6); the Dio group (dioscin-treated rats, *n* = 6); the CP group (cisplatin-treated mice, *n* = 6); and the Dio + CP group (dioscin plus cisplatin-treated rats, *n* = 6). Rats were gavaged with dioscin (60 mg/kg) for ten days, and cisplatin (10 mg/kg) was intraperitoneally injected once on the seventh day. To determine whether dioscin plays a reno-protective role through the Nrf2/HO-1 signaling pathway, 12 male Wistar rats were randomly divided into two groups: the Dio + CP group and the -N group (ML385 [Nrf2 inhibitor] + dioscin + cisplatin-treated rats). Beginning on day seven, ML385 (30 mg/kg) was intraperitoneally injected 30 min before dioscin administration in the rats once daily for three days. The schematic of the animal study design is shown in Figure 1A. The dosage and formulation methods of all drugs are based on previous reports [23,24]. After 72 h of cisplatin treatment, all animals were anesthetized using 5% isoflurane to collect the blood and then euthanized to collect renal tissues. For microscopic observation, a portion of the tissues were fixed in 4% paraformaldehyde or 2.5% glutaraldehyde phosphate. The remaining kidney tissues were rapidly quenched in liquid nitrogen and then stored at −80 °C for subsequent experiments. All procedures used in this experiment were approved by the Institutional Animal Care and Use Committee of Northeast Agricultural University (SRM-11).

### 2.2. Cell Culture and Treatment

An HK2 human kidney tubular epithelial cell line was purchased from the Procell Life Science & Technology Co., Ltd. (Wuhan, China). Cells were cultured in DMEM containing 10% fetal bovine serum and 1% penicillin/streptomycin at 37 °C in a humidified incubator with 5% carbon dioxide. In this experiment, HK2 cells were treated differently according to the following different groups: C group (control group); Dio group (dioscin group); CP group (cisplatin group); Dio + CP group (dioscin + cisplatin group); -N group (ML385 + dioscin + cisplatin group). The dosage of drugs is based on previous studies [8,23,25]. The schematic of the HK2 cells’ study design is shown in Figure 1B.

### 2.3. Transmission Electron Microscope and Hematoxylin-Eosin Staining (HE)

Rat kidney tissues fixed in 4% paraformaldehyde were used for hematoxylin-eosin (H&E) staining. The stained sections were used to observe the pathological changes of rat kidney by light microscopy. Renal tissues of rats fixed in 2.5% glutaraldehyde were used for ultrastructural observation using a transmission electron microscope (TEM; GEM-1200ES, Japan). The specific steps are consistent with our previous reports [26].

### 2.4. Biochemical Reagent Kit

The levels of blood urea nitrogen (Bun), serum creatinine (Scr), malondialdehyde (MDA), and glutathione (GSH) were evaluated using a biochemical kit (Nanjing Jiancheng Bioengineering Institute, Nanjing, China), according to the manufacturer’s instructions.

### 2.5. ROS Detection

Cryosections from frozen kidney tissues (5 μm) were prepared using a Leica CM1900 cryostat (Leica). The sections were stained with dihydroethidium (DHE) solution for 30 min in the dark at 37 °C and then washed three times with phosphate-buffered saline (PBS). Finally, a fluorescence microscope (Olympus; Tokyo, Japan) was used to photograph the section. Th detection of ROS levels in HK2 cells was via a Reactive Oxygen Species Assay Kit (Dalian Meilun, China). All steps of the detection were carried out according to the manufacturer’s instructions.

### 2.6. TUNEL Staining

Apoptotic cells were quantified using the TdT-mediated dUTP nick-end labeling (TUNEL) in situ cell death detection kit (Roche Diagnostics) according to the manufacturer’s instructions. Positive cells exhibiting green fluorescence were detected using a fluorescent microscope. The numbers of dead cells were determined as percentages of TUNEL-positive cells out of the total number of cells.

### 2.7. Cell Viability Assay

The HK2 cells were seeded into 96-well plates for 24 h to make them adhere to the wall. After being processed according to the above cell modeling method, the cell viability was detected using CCK8 reagent purchased from Dalian Meilun Bio Co., Ltd. All steps were carried out according to the instructions.

### 2.8. AO/EB Double Fluorescence Staining

The treated cells were digested with trypsin, washed with PBS, and collected by centrifugation. Cell staining was performed using an Annexin V-FITC/PI Apoptosis detection Kit (Dalian Meilun, China) according to the manufacturer’s instructions, and a fluorescence microscope (Olympus, Japan) was used to photograph them.

### 2.9. Immunohistochemistry and Immunofluorescence

Kidney tissues were fixed in 4% paraformaldehyde for 48 h, embedded in paraffin, and sliced into 5μm sections based on routine protocols. Immunohistochemical (IHC) and immunofluorescence (IF) were performed as previously described [27,28]. The positive area of CAT (Bioss; 1:100) and FSP1 (Proteintech; 1:50), and the fluorescence intensities of GPX4 (Bioss; 1:100), (Abclonal; 1:50), Nrf2 (Bioss; 1:100) and HO-1 (Proteintech; 1:50) in the photos were detected by Image J software.

### 2.10. FerroOrange Staining

HK2 cells were seeded into six-well plates and treated with different drugs, and then ferroOrange staining was performed using the FerroOrange kit purchased from Dojindo Laboratories (Japan). All steps were carried out according to the instructions.

### 2.11. Western Blot

The protein was extracted from rat kidney and HK2 cells using a protein extraction kit purchased from Wanlei Biological Co., Ltd. (Shenyang, China), and stored in liquid nitrogen for later use. The western blot assay method is consistent with our previous report [26]. In short, the protein is transferred to the PVDF membrane and combined with the corresponding primary and secondary antibody, and then the ECL luminescent solution is dropped on the membrane to obtain the protein signal through a chemiluminescence imager (Tanon, China). The relative expression levels were calculated by comparing them to the expression of the β-actin. The antibodies, dilution factors, sources and other information are presented in Table 1.

### 2.12. Statistical Analysis

A statistical analysis of all data was conducted using GraphPad Prism version 8.0 software. All results were expressed as the mean ± SD. Statistical significance was obtained by one-way ANOVA or unpaired Student’s *t*-test using Tukey’s post hoc test. *p* values of less than 0.05 were considered statistically significant. The software showed a normal distribution.

## 3. Results

### 3.1. Dioscin Relieves Cisplatin-Induced AKI

To explore the effect of dioscin on cisplatin-induced AKI in rats, the pathological and ultrastructural changes of rat kidneys were observed by H&E staining and TEM. The results were shown in Figure 1C. After cisplatin treatment, the epithelial cells of rat renal tubules were degenerated (blue arrow) and part of them sloughed off into the lumen (black arrow). In addition, abnormal nuclei (red arrow) and inflammatory cell infiltrated lightly (yellow arrow) were also observed in rat kidneys. Disoscin pretreatment improved the pathological changes of rat kidney. The TEM results showed that dioscin markedly reduced cisplatin-induced mitochondrial damage (red pentagram) in rat kidneys (Figure 1C). Furthermore, the levels of renal function indicators including blood urea nitrogen (BUN) and serum creatinine (SCr) in rats were detected, and the results showed that dioscin significantly alleviated the increase of BUN and SCr caused by cisplatin (Figure 1D,E) (*p* < 0.05). The results of a CCK8 assay showed that compared with the CP group, the HK2 cells activity in the Dio + CP group was significantly increased (Figure 1F) (*p* < 0.01). These results suggest that dioscin has a positive effect on cisplatin-induced AKI.

### 3.2. Dioscin Ameliorates Oxidative Damage in Rat Kidneys and HK2 Cells

**Figure 2:**
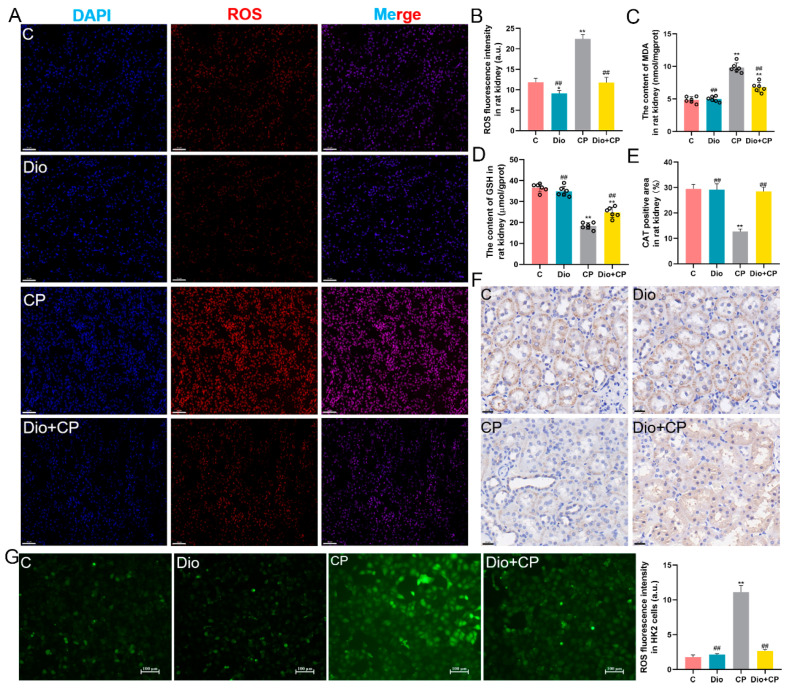
Dioscin ameliorates oxidative damage in rat kidneys and HK2 cells. (**A**,**B**) Representative images (scale bar = 50 μm) and quantification of ROS (red) in rat kidney tissue (*n* = 5). (**C**,**D**) Levels of MDA and GSH in rat kidney tissue (*n* = 6). (**E**,**F**) Representative images (scale bar = 20 μm) and quantification of CAT immunohistochemistry in rat kidney (*n* = 5). (**G**) Representative images (scale bar = 100 μm) and quantification of ROS (green) in HK2 cells (*n* = 5). C, control group; Dio, dioscin group; CP, cisplatin group; Dio + CP, dioscin + cisplatin group. Results are presented as Mean ± SD. Statistical significance was obtained by one-way ANOVA. ** *p* < 0.01 compared with C group. ^##^ *p* < 0.01 compared with CP group.

To explore the effect of dioscin on cisplatin-induced oxidative damage in rat kidneys, the levels of ROS, MDA, GSH and CAT were detected. The results showed that after cisplatin treatment, the oxidation products ROS and MDA accumulated in rat kidneys, and dioscin significantly alleviated this change (Figure 2A–C) (*p* < 0.01). Furthermore, the detection results of GSH showed that compared with the CP group, the GSH level in the kidneys of the Dio + CP group was remarkedly increased (Figure 2D) (*p* < 0.01). Moreover, the immunohistochemical results showed that CAT levels in the kidneys of rats were significantly decreased after cisplatin treatment, and this change was reversed by dioscin (Figure 2E,F) (*p* < 0.01). The in vitro test results showed that compared with the C group, the level of ROS in the CP group was evidently increased, while dioscin significantly inhibited the accumulation of ROS in HK2 cells (Figure 2G) (*p* < 0.01).

### 3.3. Dioscin Reduces Cisplatin-Induced Apoptosis in Rat Kidneys and HK2 Cells

**Figure 3:**
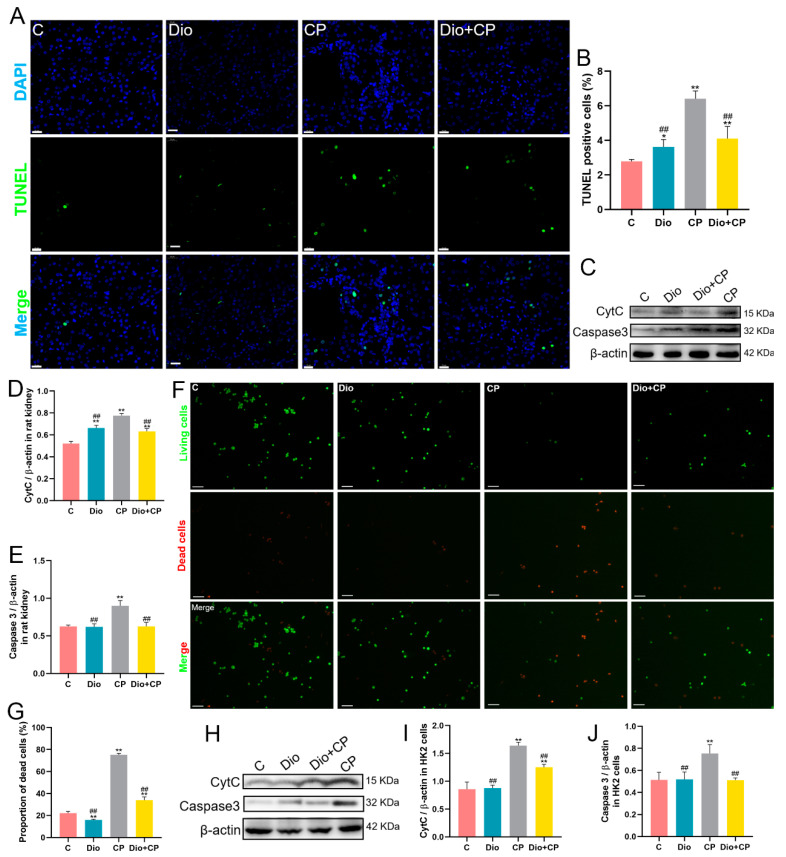
Dioscin reduces cisplatin-induced apoptosis in rat kidney and HK2 cells. (**A**,**B**) Representative images and quantification of TUNEL staining (scale bar = 20 μm) in rat kidney (*n* = 5). (**C**–**E**) The protein expression of CytC and Caspase3 in rat kidney (*n* = 3). (**F**,**G**) Representative images and quantification of AO/EB staining (scale bar = 100 μm) in HK2 cells (*n* = 5). (**H**–**J**) The protein expression of CytC and Caspase3 in HK2 cells (*n* = 3). C, control group; Dio, dioscin group; CP, cisplatin group; Dio + CP, dioscin + cisplatin group. Results are presented as Mean ± SD. Statistical significance was obtained by one-way ANOVA. * *p* < 0.05, ** *p* <0.01 compared with C group. ^##^ *p* < 0.01 compared with CP group.

Mitochondrial damage is a representative feature of apoptosis, and dioscin markedly improved the mitochondria destroyed by cisplatin. In order to determine the effect of dioscin on cisplatin-induced renal cell apoptosis, we performed TUNEL staining on rat kidney and found that compared with the CP group, the apoptotic cells in the Dio + CP group were significantly reduced (Figure 3A,B) (*p* < 0.01). In addition, western blot results showed that cisplatin dramatically upregulated the expression of the pro-apoptotic proteins CytC and Caspase 3 in rat kidney, while dioscin mitigated this change (Figure 3C–E) (*p* < 0.01). In in vitro experiments, we stained HK2 cells with an AO/EB assay and found that compared with the CP group, the dead cells (red) in the Dio + CP group were memorably decreased and the viable cells (green) were significantly increased (Figure 3F,G) (*p* < 0.01). Furthermore, the levels of pro-apoptotic proteins in HK2 cells were also detected, and the results were consistent with in vivo experiments, that is, dioscin substantially reduced the cisplatin-induced the protein expression of CytC and Caspase 3 (Figure 3H–J) (*p* < 0.01).

### 3.4. Dioscin Improves Cisplatin-Induced Ferroptosis in Rat Kidneys and HK2 Cells

**Figure 4:**
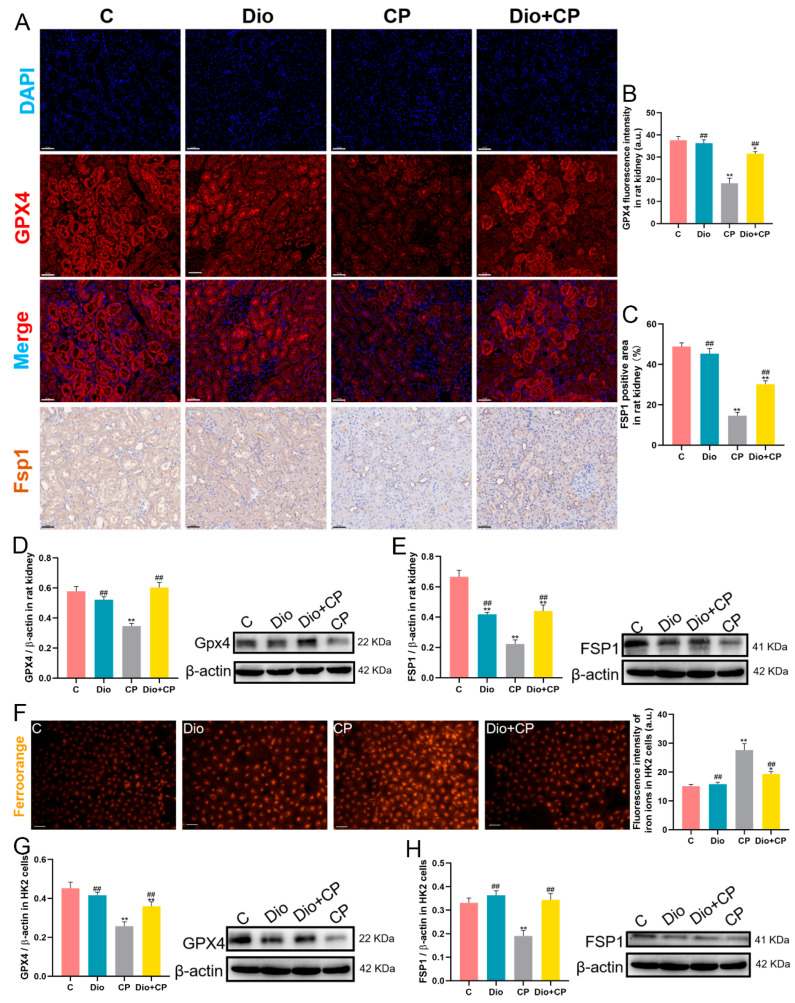
Dioscin improves cisplatin-induced ferroptosis in rat kidney and HK2 cells. (**A**–**C**) Representative images and quantification of GPX4 immunofluorescence (scale bar = 50 μm) and FSP1 immunohistochemistry (scale bar = 50 μm) in rat kidneys (*n* = 5). (**D**,**E**) The protein expressions of GPX4 and FSP1 in rat kidneys (*n* = 3). (**F**) Representative images and quantification of ferro orange staining (scale bar = 50 μm) in HK2 cells (*n* = 5). (**G**,**H**) The protein levels of GPX4 and FSP1 in HK2 cells (*n* = 3). C, control group; Dio, dioscin group; CP, cisplatin group; Dio + CP, dioscin + cisplatin group. Results are presented as Mean ± SD. Statistical significance was obtained by one-way ANOVA. * *p* < 0.05, ** *p* < 0.01 compared with C group. ^##^ *p* < 0.01 compared with CP group.

Lipid peroxidation is the main reason for inducing ferroptosis, and dioscin effectively inhibits the level of the lipid peroxidation product MDA. In order to explore the effect of dioscin on ferroptosis, we detected the levels of ferroptosis representative proteins GPX4 and FSP1 in rat kidneys by immunofluorescence and immunofluorescence, respectively. The results showed that cisplatin memorably weakened the fluorescence intensity of GPX4 and reduced the positive area of FSP1 in rat kidneys, while the levels of GPX4 and FSP1 were significantly enhanced in the Dio + CP group compared with the CP group (Figure 4A–C) (*p* < 0.01). Moreover, the results obtained by detecting the protein levels of GPX4 and FSP1 by Western Blot are consistent with the above results (Figure 4D,E) (*p* < 0.01). In vitro, FerroOrange staining was used to detect Fe^2+^ in HK2 cells, and the results showed that intracellular Fe^2+^ levels (orange) were significantly enhanced after cisplatin treatment, while dioscin remarkedly arrested this change (Figure 4F) (*p* < 0.01). In addition, the detection results of ferroptosis-related protein levels in HK2 cells showed that the protein expressions of GPX4 and FSP1 in the Dio + CP group were substantially increased compared to the CP group, which was consistent with the in vitro results (Figure 4G,H) (*p* < 0.01). These findings suggest that dioscin attenuates cisplatin-induced renal ferroptosis.

### 3.5. Dioscin Upregulates Nrf2/HO-1 Signaling in Cisplatin-Treated Rat Kidney and HK2 Cells

**Figure 5:**
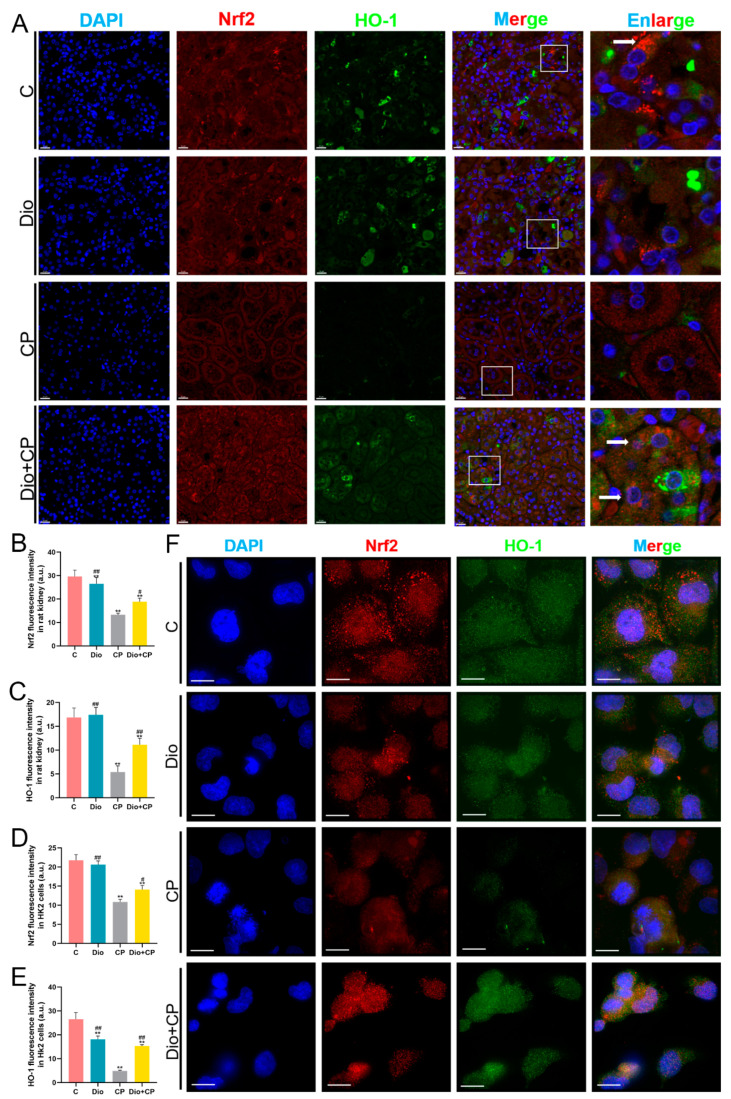
Dioscin upregulates Nrf2/HO-1 signaling in cisplatin-treated rat kidney and HK2 cells. (**A**–**C**) Representative images and quantification of Nrf2 and HO-1 immunofluorescence (scale bar = 20 μm) in rat kidneys (*n* = 5). (**D**–**F**) Representative images and quantification of Nrf2 and HO-1 immunofluorescence (scale bar = 15 μm) in HK2 cells (*n* = 5). C, control group; Dio, dioscin group; CP, cisplatin group; Dio + CP, dioscin + cisplatin group. Results are presented as Mean ± SD. Statistical significance was obtained by one-way ANOVA. ** *p* < 0.01 compared with C group. ^#^ *p* < 0.05, ^##^ *p* < 0.01 compared with CP group.

To explore whether dioscin exerts a protective effect on the kidney through the Nrf2/HO-1 pathway, we detected Nrf2 and HO-1 levels by double immunofluorescence. As shown in Figure 5A, the levels of Nrf2 (red) and HO-1 (green) markedly weakened after cisplatin treatment, while both of the levels in the Dio + CP group were significantly higher than those in the CP group (Figure 5A–C) (*p* < 0.01). Moreover, it is obvious that Nrf2 is abundantly present in the cytoplasm (white arrow) in the C group, and Nrf2 is translocated to the nucleus (white arrow) in the Dio + CP group (Figure 5A). The same results were also seen in in vitro experiments, suggesting that the protective effect of dioscin on the kidney is related to Nrf2/HO-1 signaling.

### 3.6. The Protective Effect of Dioscin on Rat Kidneys Is Attenuated after Nrf2 Inhibition

**Figure 6:**
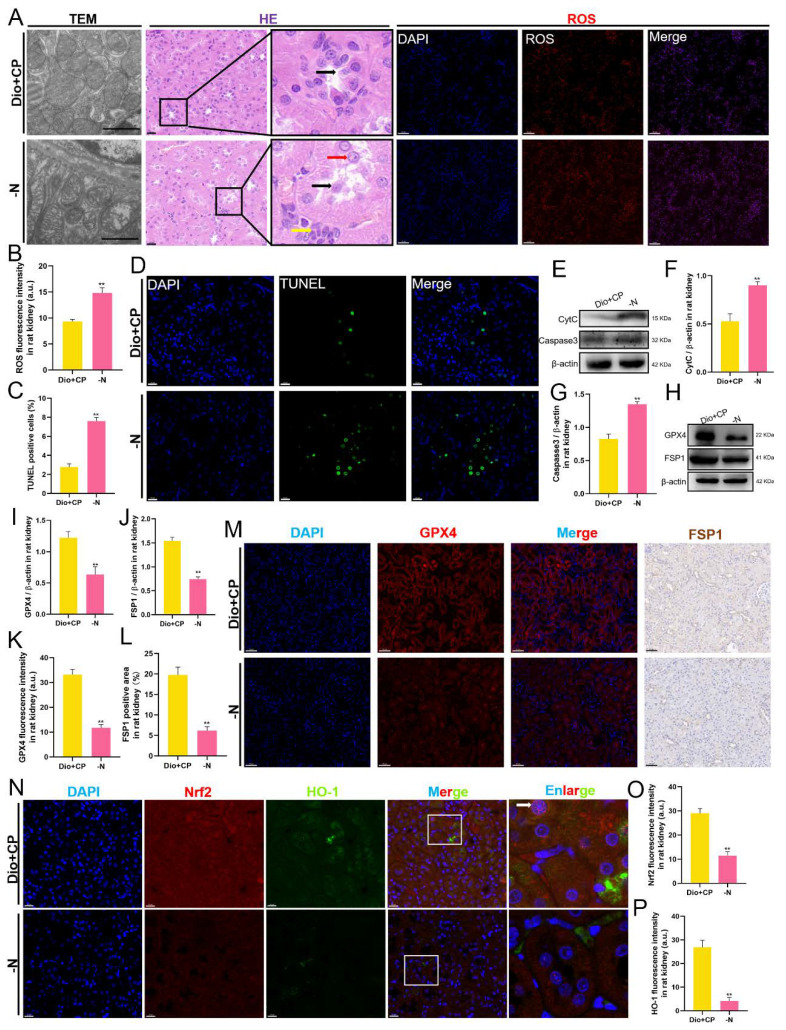
The protective effect of dioscin on rat kidneys is attenuated after Nrf2 inhibition. (**A**) Representative images of transmission electron microscope (scale bar = 1 μm), H&E staining (scale bar = 20 μm), and ROS staining (scale bar = 50 μm) in rat kidneys (*n* = 5). (**B**) Quantification of ROS staining in rat kidneys. (**C**,**D**) Representative images and quantification of TUNEL staining (scale bar = 20 μm) in rat kidneys (*n* = 5). (**E**–**G**) The protein levels of CytC and Caspase3 in rat kidneys (*n* = 3). (**H**–**J**) The protein levels of GPX4 and FSP1 in rat kidneys (*n* = 3). (**K**–**M**) Representative images and quantification of GPX4 immunofluorescence (scale bar = 50 μm) and FSP1 immunohistochemistry (scale bar = 50 μm) in rat kidneys (*n* = 5). (**N**–**P**) Representative images and quantification of Nrf2 and HO-1 immunofluorescence (scale bar = 20 μm) in rat kidneys (*n* = 5). C, control group; Dio, dioscin group; CP, cisplatin group; Dio + CP, dioscin + cisplatin group. ** *p* < 0.01 represents extremely significant difference. Results are presented as Mean ± SD. Statistical significance was obtained via an unpaired Student’s *t*-test.

To further confirm that dioscin exerts a nephroprotective effect though Nrf2/HO-1 signaling, we suppressed Nrf2 expression in Dio + CP group rats using ML385 and performed identical experiments as above. As shown in Figure 6, compared with the Dio + CP group, the renal mitochondrial damage of rats in the -N group was aggravated, and the renal tubular epithelial cells were shed and accompanied by inflammatory cells infiltration (Figure 6A). In addition, the level of ROS in the kidneys of rats in the -N group was significantly higher than that in the Dio + CP group (Figure 6A,B) (*p* < 0.01). The results of TUNEL staining showed that compared with the Dio + CP group, the apoptotic cells in the kidneys of the rats in the -N group increased significantly (Figure 6C,D) (*p* < 0.01). Furthermore, the expression of pro-apoptotic proteins including CytC and Caspase 3 in rat kidneys was markedly increased after Nrf2 inhibition (Figure 6E–G) (*p* < 0.01). Furthermore, the western blot showed that the expression of anti-ferroptosis related proteins including GPX4 and FSP1 in the -N group were memorably lower than that in Dio + CP group (Figure 6H–J) (*p* < 0.01). GPX4 fluorescence intensity and the FSP1 positive area in rat kidneys were both reduced after Nrf2 inhibition (Figure 6K–M) (*p* < 0.01). In addition, the double immunofluorescence results of Nrf2 and HO-1 showed that compared with the Dio + CP group, the levels of Nrf2 and HO-1 in the -N group decreased significantly (Figure 6N–P) (*p* < 0.01). These results suggest that blocking the Nrf2/HO-1 signaling attenuates the nephroprotective effect of dioscin in rats.

### 3.7. The Protective Effect of Dioscin on HK2 Cells Is Attenuated after Nrf2 Inhibition

**Figure 7:**
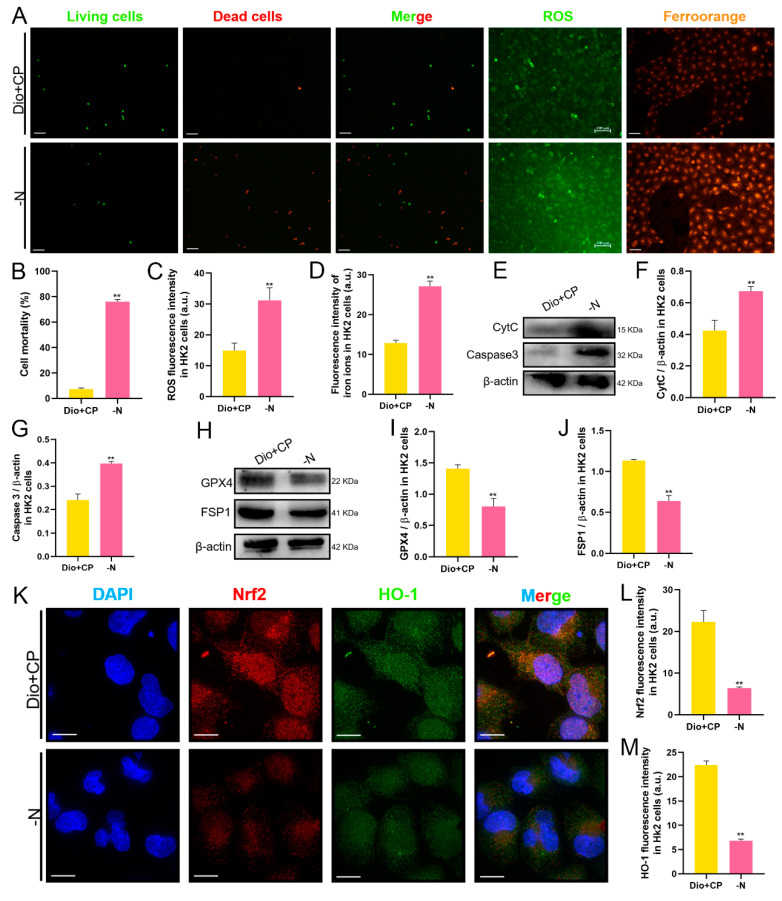
The protective effect of dioscin on HK2 cells is attenuated after Nrf2 inhibition. (**A**–**D**) Representative images and quantification of AO/EB staining (scale bar = 100 μm), ROS staining (scale bar = 100 μm) and ferro orange staining (scale bar = 50 μm) in HK2 cells (*n* = 5). (**E**–**G**) The protein expressions of CytC and Caspase3 in HK2 cells (*n* = 3). (**H**–**J**) The protein expressions of GPX4 and FSP1 in HK2 cells (*n* = 3). (**K**–**M**) Representative images and quantification of Nrf2 and HO-1 immunofluorescence (scale bar = 15 μm) in HK2 cells (*n* = 5). C, control group; Dio, dioscin group; CP, cisplatin group; Dio + CP, dioscin + cisplatin group. ** *p* < 0.01 represents extremely significant difference. Results are presented as Mean ± SD. Statistical significance was obtained by an unpaired Student’s *t*-test.

In vitro, we detected cell death by AO/EB staining, and also detected the levels of ROS and Fe^2+^ in HK2 cells. The results are shown in Figure 7. Compared with the Dio + CP group, the number of dead cells (red) in the -N group was significantly increased, while the viable cells (green) were markedly reduced (Figure 7A,B). Furthermore, the levels of both ROS (Figure 7A,C) and Fe^2+^ (Figure 7A,D) in HK2 cells in the -N group were higher than those in the Dio + CP group (*p* < 0.01). In addition, after Nrf2 inhibition, the expression of pro-apoptotic proteins including CytC and Caspase 3 were evidently enhanced in HK2 cells (Figure 7E–G), while the levels of anti-ferroptosis related proteins including GPX4 and FSP1 were significantly decreased (Figure 7H–J) (*p* < 0.01). The results of cellular immunofluorescence showed that compared with the Dio + CP group, the fluorescence signals of Nrf2 (red) and HO-1 (green) in HK2 cells in the -N group were remarkably weakened (Figure 7K–M) (*p* < 0.01). These findings further clarify that Nrf2/HO-1 signaling plays a key role in dioscin attenuating cisplatin-induced AKI.

## 4. Discussion

**Figure 8:**
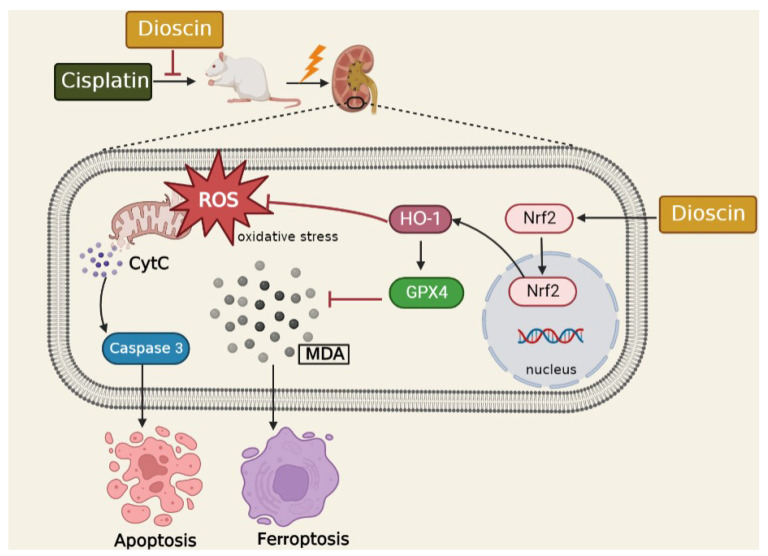
The schematic diagram of regulatory of Dioscin in the Nrf2/HO-1signaling to improve cisplatin-induced kidney injury. Cisplatin increases the accumulation of ROS and MDA and the levels of CytC and Caspase3 as well as reduces levels of GPX4 and FSP1 in kidneys, resulting in oxidative stress, apoptosis and ferroptosis in renal tissue. Dioscin exerts a protective effect on the kidney by upregulating Nrf2/HO-1 signaling.

AKI is a common complication of hospitalized critically ill patients, the main treatment method is fluid support and agents to prevent hemodynamic changes [29], and there is no targeted prevention method. Dioscin naturally exerts in Dioscorea plants such as Dioscorea nipponica makino and Dioscorea zingiberensis, and has been found to have antioxidant and anti-inflammatory effects. In the current study, cisplatin-administered rats and HK2 cells were pretreated with dioscin to observe the effect of dioscin on AKI and explore the exact molecular mechanism. Our results showed that dioscin upregulated the Nrf2/HO-1 signaling pathway in vivo and in vitro and remarkably reduced oxidative stress, apoptosis and ferroptosis. However, the reno-protective effect of dioscin was weakened considerably in Nrf2 inhibited in vivo and in vitro, further suggesting that dioscin protects the kidney through the Nrf2/HO-1 signaling pathway (Figure 8).

All living organisms exhibit a dynamic balance between the oxidation and anti-oxidation process, and oxidative stress occurs when the antioxidant capacity is weakened and oxides are not efficiently scavenged. The accumulation of ROS and lipid peroxidation products (such as MDA) in renal tissues, and restricted antioxidant substances (such as SOD and GSH), are believed to be the main mechanisms of cisplatin-induced AKI. Studies have shown that free radical scavengers, such as edaravone and some reducing nutrients including vitamin C and vitamin E, can effectively protect against AKI [30,31]. Here, we found that dioscin plays a renal protective role in cisplatin-induced AKI by stimulating the antioxidant system by increasing the GSH and CAT levels to reduce the contents of ROS and MDA in the renal tissues and HK2 cells. In addition, numerous ROS accumulation can damage mitochondria and lead to the release of apoptotic factors, whereas the use of ROS blockers distinctly reduced the release of pro-apoptotic genes, including CytC and Cas3, thereby decreasing the rate of apoptosis [32]. Furthermore, many studies have shown that lowering renal apoptosis by suppressing the expression of pro-apoptotic factors such as Caspase 3 and promoting the expression of anti-apoptotic factors is an effective means to alleviate AKI [33]. Consistent with the findings of previous studies, we found that dioscin dramatically reduced the expression of pro-apoptotic proteins including CytC and Caspase 3 in rat kidneys and HK2 cells, indicating that dioscin plays a protective role in thwarting renal apoptosis.

MDA is the product of lipid peroxidation, which can induce ferroptosis. Recently, the detrimental aspect of iron, in that ferroptosis may be the trigger for tissue damage, has gradually been revealed. In a rat model of adriamycin-induced cardiomyopathy, myocardial tissue has attenuated antioxidant capacity and ferroptosis [34]. Although the signaling pathway of ferroptosis has not been fully elucidated, many studies have shown that ferroptosis is ultimately caused by directly or indirectly affecting the activity of GPX4, reducing the antioxidant capacity of cells [5,35,36]. GPX4 is considered to be the main defense mechanism of ROS-mediated membrane peroxides because of its special structure that interacts with membrane phospholipids [37,38]. As a substrate of GPX4, the insufficient supply of GSH will directly affect the function of GPX4 and lead to ferroptosis [39]. In other words, increasing GSH content and GPX4 activity can effectively alleviate ferroptosis. Additionally, Bersuker et al. first found in 2019 that the gene Fsp1 also strongly inhibits ferroptosis and operates in parallel with the canonical GSH-dependent GPX4 pathway [7]. Over the past decade, there has been increasing evidence that iron-induced toxicity is associated with multiple pathological mechanisms. Therefore, it has become increasingly clear that the alleviation of ferroptosis is an effective means to protect various tissues. In this study, we examined the levels of GPX4 and FSP1 in rat kidneys by different methods such as immunofluorescence or immunohistochemistry, and found that both of them were significantly decreased after cisplatin treatment, while dioscin markedly improved this situation. The results obtained in vitro are consistent with those in vivo, which indicates that dioscin effectively arrests cisplatin-induced ferroptosis in kidney.

The Nrf2/HO-1 signaling pathway is a cellular mechanism to counteract ROS and prevent oxidative injury. Nrf2 is a transcription factor that is ubiquitinated and rapidly degraded in the cytoplasm under normal physiological conditions [40]. When cells are attacked by ROS, Nrf2 is rapidly translocated into the nucleus to combine with the corresponding antioxidant response elements (ARE) and then trigger the transcription of target genes to play an antioxidant role. Moreover, recent studies have shown that the activation of Nrf2 appears to be resistant to ferroptosis because it promotes the expression of anti-ferroptotic genes (such as GPX4) [41]. HO-1 is an important intracellular antioxidant enzyme regulated by Nrf2, which can scavenge ROS to resist oxidative damage. Moreover, many studies have showed that Nrf2/HO-1 signaling also exerts an anti-inflammatory and anti-apoptosis effect to varying degrees [42,43]. Recently, the effect of Nrf2/HO-1 in ferroptosis has gradually been revealed. In a rat model of type 2 diabetic osteoporosis, melatonin suppresses ferroptosis and enhances cellular osteogenic capacity via activating the Nrf2/HO-1 signaling pathway [44]. Nevertheless, Feng et al. showed that ferroptosis aggravated kidney damage was associated with the upregulation of HO-1 [45]. Hence, it is necessary to explore how dioscin regulates Nrf2/HO-1 signaling in cisplatin-induced AKI, especially in renal cell ferroptosis. In the current study, we found that dioscin promotes Nrf2 entry into the nucleus in AKI rat kidney and HK2 cells, and upregulates downstream HO-1 levels. However, after inhibiting the expression of Nrf2 in rats and HK2 cells, the antioxidant, anti-apoptosis and anti-ferroptosis abilities of dioscin were dramatically attenuated, indicating that the protective effect of dioscin on cisplatin-induced AKI is closely related to the up-regulation of Nrf2/HO-1 signaling.

## 5. Conclusions

The current study illustrated that dioscin markedly alleviates AKI by thwarting renal oxidative stress, apoptosis and ferroptosis in kidney by activating the Nrf2/HO-1 signaling pathway. Our study provides new experimental data and potential therapeutic targets for the prevention and treatment of AKI. Although the detailed mechanism of the role of Nrf2/HO-1 in ferroptosis remains controversial, our findings contribute to the further understanding of the regulation and function of Nrf2/HO-1 in renal ferroptosis.

## Figures and Tables

**Table 1:** Western Blot Antibody Information.

Antibody	Catalog Number	Dilution Ratio	Manufacturer	Molecular Weight
CytC	WL02410	1:1000	Wanlei	15 KDa
Caspase3	WL04004	1:500	Wanlei	32 KDa
GPX4	A1933	1:500	Abclonal	22 KDa
Fsp1	20886-1-AP	1:500	Proteintech	41 KDa
β-actin	bs-0061R	1:500	Bioss	42 KDa

## Data Availability

All data supporting the findings during this study are available in this manuscript.
